# *Clostridium butyricum*: a promising approach to enhancing intestinal health in poultry

**DOI:** 10.3389/fvets.2025.1544519

**Published:** 2025-05-21

**Authors:** Xiaopeng Tang, Yan Zeng, Meijun Li

**Affiliations:** ^1^State Engineering Technology Institute for Karst Desertification Control, School of Karst Science, Guizhou Normal University, Guiyang, China; ^2^Key Laboratory for Information System of Mountainous Areas and Protection of Ecological Environment, Guizhou Normal University, Guiyang, China; ^3^College of Animal Science and Technology, Hunan Biological and Electromechanical Polytechnic, Changsha, China

**Keywords:** *Clostridium butyricum*, growth performance, intestinal health, gut microbiota, poultry

## Abstract

The intestinal tract is essential for the overall health and productivity of animals, including poultry. *Clostridium butyricum* (*C. butyricum*) is a probiotic bacterium that has been shown to be a promising candidate for improving intestinal function and subsequently optimizing poultry growth. The beneficial effects of *C. butyricum* on intestinal health can be attributed to several key mechanisms. First, it helps maintain the balance of the intestinal microbiota by inhibiting the growth of harmful bacteria and promoting the proliferation of beneficial bacteria. This microbial homeostasis is essential for efficient nutrient digestion and absorption. Second, *C. butyricum* enhances the integrity of the intestinal barrier. It enhances the integrity of epithelial tight junctions, reducing the permeability of the intestinal mucosa and preventing the invasion of pathogenic substances. Furthermore, *C. butyricum* participates in the regulation of immune responses within the intestinal environment. It stimulates the production of immunoglobulins and cytokines, enhancing the immune defense mechanisms of the host. Additionally, *C. butyricum* influences the metabolism of nutrients in the intestine. It promotes the synthesis of short-chain fatty acids (SCFAs), which provide an energy source for intestinal cells and contribute to maintaining a healthy intestinal environment. Intestinal health is the basis of animal growth, and *C. butyricum* ultimately enhances production performance in poultry by regulating intestinal health. Studies have demonstrated that the administration of *C. butyricum* leads to improved feed conversion efficiency, increased weight gain, and enhanced overall production performance in poultry. However, further research is still needed to fully elucidate the complex interactions and precise molecular mechanisms underlying its beneficial effects. Understanding these mechanisms in detail will not only provide important insights for improving poultry production efficiency but also contribute to the development of more effective and sustainable strategies in the poultry industry.

## Introduction

Poultry husbandry not only serves as a valuable source of protein for human consumption but also plays a crucial role in agricultural production, contributing significantly to agricultural economics and food security (1). Consequently, enhancing the efficiency of poultry production is vital for enhancing yields, lowering costs, and promoting sustainability. Current strategies to improve the efficiency of poultry production include genetic improvement and nutritional management (2, 3). Among these, the pivotal role of nutritional strategies in maximizing poultry productivity and promoting poultry health has received significant attention, as it addresses the increasing consumer demand for higher quality and safer food products (4, 5).

The intestinal tract of poultry is essential for nutrient digestion, absorption, and defense against pathogens (6). The intestinal health of poultry is closely associated with their growth, as a healthy gut is essential for enabling poultry to fully exploit nutrients in feedstuffs while maintaining normal growth patterns alongside resisting disease invasion (7–9). Therefore, maintaining the intestinal health of poultry is very important to improve the efficiency of poultry production. However, various factors including feed quality, housing environment conditions, and stress can all contribute to microbiota imbalance within the gut, malfunctioning intestinal barriers, and abnormal morphological changes within the intestines of poultry, resulting in adverse effects on their health and growth performance (10–13). Antibiotics have traditionally been used as growth promoters and gut health regulators in poultry. However, in recent years, with increasing emphasis on food safety and environmental protection, the pressing need for alternatives to antibiotics in livestock and poultry farming has emerged due to issues stemming from antibiotic misuse such as antibiotic resistance emergence, drug residues, and environmental pollution (14–16).

Probiotics, beneficial microorganisms, show significant potential in livestock and poultry farming (17–22). Previous studies have demonstrated that probiotics can inhibit bacterial infection, in-crease antioxidant capacity, alleviate toxicity and improve intestinal health in poultry production (23–26). The probiotics used in poultry production mainly include *Bacillus* (27), *Lactobacillus* (28), *Bifidobacterium* (29), and *Enterococcus* (30). Among these, *Bacillus* is more advantageous in regulating intestinal health and enhancing the growth performance of poultry as an alternative to antibiotics, due to its ability to produce resistance endospores, which confer heat resistance during feed granulation, low pH resistance in the stomach, and storage stability at ambient temperatures (22, 31, 32). *Clostridium butyricum* (*C. butyricum*) is a gram-positive obligate anaerobic bacterium belonging to *Bacillus* family and *Clostridium* genus that can produce butyric acid and lactic acid. It has the characteristics of *Bacillus* that can produce spores, making it resistant to heat, stomach acid, and bile salts, essential for its application in feed production (17, 33). *Clostridium butyricum* has attracted significant attention as a crucial probiotic due to its role in preserving gut health and enhancing the growth performance of poultry (23, 27, 34–36). This is attributed to the ability of *C. butyricum* to modulate intestinal flora balance and bolster immune function, while enhances nutrient absorption by producing diverse enzymes and vitamins (23, 36–38), thereby improving the growth performance of poultry. With its distinct biological characteristics and beneficial functions, *C. butyricum* has emerged as a potential alter-native to antibiotics.

However, Differences in digestion among poultry species, such as chickens, ducks, and geese, may influence the effects and mechanisms of *C. butyricum*. Therefore, although the application of *C. butyricum* in poultry production has received extensive attention, it is necessary to review its application effects on different poultry species. In this context, it is of great theoretical and practical significance to conduct indepth research on the effects of *C. butyricum* on the health of poultry intestines and its role in promoting growth performance. By understanding how *C. butyricum* works, we can offer more scientific and rational breeding strategies for the poultry sector, reducing reliance on antibiotics, enhancing breeding efficiency, and ensuring the quality and safety of poultry products to meet consumer demand for healthy food. This review aims to summarize the current understanding of how *C. butyricum* benefits poultry growth performance by regulating intestinal health.

## *Clostridium butyricum* promotes intestinal health of poultry

The intestinal health of poultry is a complex and critical aspect of poultry production. A healthy intestinal system is essential for efficient nutrient digestion and absorption, immune function, and pathogen resistance (39). In actual production, poultry often face a variety of stressors, including heat stress (40), *Eimeria* spp. infection (41), *Clostridium perfringens* (*C. perfringens*) infection (42), mycotoxins (43), and other harmful effects, which can damage the structure of intestinal mucosa and intestinal barrier function. In recent years, the use of probiotics, such as *C. butyricum*, has gained attention as a potential strategy to enhance intestinal health and improve the performance of poultry (35–38).

### *Clostridium butyricum* promotes intestinal development

*Clostridium butyricum* has been shown to have positive effects on the morphology and integrity of the intestinal mucosa in poultry including broiler chickens (38), laying hens (44), ducks (36), and goose (45). It promotes the development of villi, increases the intestinal villus height (VH), decreases the crypt depth (CD), and increases the VH-to-CD ratio (VCR) (38, 45–47). For instance, Liu et al. (36) showed that dietary *C. butyricum* supplementation significantly increased intestinal VH of Pekin ducks at 42 days. Obianwuna et al. (44) showed that dietary addition of *C. butyricum* (*C. butyricum* zlc-17, and *C. butyricum* lwc-13) significantly increased the VH, villus width, surface area, and VCR, and significantly reduced the CD of laying hens. Yu et al. (45) showed that dietary supplemented with *C. butyricum* significantly increased duodenal weight and absolute weight (weight/length), and significantly increased ileum VH of geese. Xu et al. (46) showed that *C. perfringens* destroys intestinal structure and deepens intestinal CD in broilers, while *C. butyricum* can improve intestinal morphological structure and decrease the CD. These structural improvements enhance the absorptive capacity of the intestine and facilitate better nutrient uptake.

The promoting effect of *C. butyricum* on intestinal morphology is primarily attributed to its strong capacity for butyric acid production, which serves as a direct energy source for the growth of intestinal villi, plays a pivotal nutritional role in the development of intestinal villi, and facilitates the repair of damaged intestinal epithelial cells (17, 48). Additionally, it is associated with its strong ability to promote the secretion of intestinal digestive enzymes, such as alkaline phosphatase, amylase, protease, trypsin, and lipase (38, 49, 50), which assist in the degradation of starch, fat, protein and anti-nutrient factors, and thus improve the digestion and absorption efficiency of nutrients. Therefore, the addition of *C. butyricum* to poultry diets can positively impact the development of the intestinal tract in poultry by enhancing intestinal morphology and structure, as well as stimulating the secretion of digestive enzymes. The inclusion of *C. butyricum* in poultry diets may therefore lead to overall enhancements in poultry health and productivity.

### *Clostridium butyricum* enhances intestinal antioxidant capacity

Oxidative stress is a state of imbalance between the overproduction of reactive oxygen species (ROS) within the body and an inadequate function of the antioxidant defense system (51–53). ROS, such as superoxide anion, hydrogen peroxide, and hydroxyl radical, can cause lipid peroxidation, protein oxidation, and DNA damage (54). Malonaldehyde (MDA) is the main product of lipid peroxidation, protein carbonyl (PCO) is a marker of oxidative damage of protein, and 8-hydroxy-2 ‘-deoxyguanosine (8-OHdG) is a marker of oxidative damage of nucleic acid, which are generally considered as the markers of oxidative damage (55, 56). To counteract the harmful effects of ROS, cells possess a complex antioxidant defense system. This includes enzymatic antioxidants such as superoxide dismutase (SOD), catalase (CAT), and glutathione peroxidase (GSH-Px), which convert ROS into less reactive species. Non-enzymatic antioxidants like vitamins C and E, glutathione, and carotenoids scavenge ROS and prevent their accumulation (57). The balance between ROS generation and antioxidant defense is critical for maintaining cellular homeostasis.

*Clostridium butyricum* is a beneficial bacterium that has been shown to exert multiple beneficial effects, including antioxidant function on the intestinal of poultry (45, 50). Studies have demonstrated that supplementation with *C. butyricum* can reduce the levels of oxidative stress markers in the intestinal tissue of poultry. For example, Xiang et al. (58) demonstrated that feeding a diet containing *C. butyricum* reduced ROS levels in the ileum (4.12 U/mL vs. 3.57 U/mL) and cecum (3.81 U/mL vs. 3.08 U/mL), and serum MDA levels (16.82 nmol/mL vs. 13.40 nmol/mL) of laying hens. Yu et al. (45) showed that the combination of *C. butyricum* and *Bacillus subtilis* (*B. subtilis*) significantly increased SOD levels in jejunum and T-AOC and GSH-Px levels in ileum of geese. Li et al. (50) demonstrated that supplementation of *C. butyricum* to broilers led to a significant increase in serum SOD (83.85 U/mL vs. 90.90 U/mL at 21 days; 117.2 U/mL vs. 128.1 U/mL at 42 days) and total antioxidant capacity (T-AOC; 0.68 U/mL vs. 0.71 U/mL at 42 days), as well as a notable reduction in MDA levels (4.15 nmol/mL vs. 3.67 nmol/mL at 42 days). Additionally, there was a significant upregulation in the mRNA expression of nuclear factor erythroid 2-related factor 2 (*Nrf2*) and *SOD1* in the jejunum mucosa (50). Nrf2 is a kind of transcription factor that closely related to cellular redox balance, which helps cells remain stable in response to oxidative stress. Under normal conditions, Nrf2 binds with Kelch-like ECH-associated protein 1 (Keap1) in the cytoplasm, where Keap1 promotes the ubiquitination and degradation of Nrf2 by the proteasome (51). When cells experience oxidative stress, ROS modify cysteine residues on Keap1, causing Nrf2 to dissociate from Keap1. The released Nrf2 then enters the nucleus, forms a heterodimer with small Maf proteins, and binds to the antioxidant response elements (ARE) in the promoter regions of target genes, thereby activating the transcription of downstream antioxidant genes, such as phase II metabolic enzymes (Heme Oxygenase-1 (HO-1) and NAD(P)H:Quinone Oxidoreductase 1 (NQO1)), and antioxidant enzymes (such as SOD, CAT, and GSH-Px) (51, 59). These antioxidant enzymes can eliminate ROS, reduce oxidative damage and maintain the redox balance of cells. It means that *C. butyricum* alleviates intestinal oxidative damage in poultry by activating the Nrf2 signaling pathway. *Clostridium butyricum* enhances intestinal mucosal barrier function.

The intestinal mucosal barrier in poultry is a complex and dynamic structure composed of multiple layers and components. The epithelial layer, which consists of enterocytes, goblet cells, and Paneth cells, forms the first line of defense (39). Tight junctions between enterocytes regulate the paracellular passage of substances and maintain the integrity of the physical barrier (22). The mucus layer secreted by goblet cells acts as a chemical barrier and traps pathogens (22). Therefore, the intestinal mucosal barrier, composed of a physical barrier and a chemical barrier, is very important for intestinal health. In production practice, poultry is susceptible to pathogens, such as *Escherichia coli* (*E. coli*) (38), *Salmonella* (60), and *C. perfringens* (46, 61), which can increase intestinal permeability and damage the intestinal mucosal barrier, resulting in significant economic losses in poultry farming. For instance, Zhang et al. (38) demonstrated that broilers exhibited a significant increase in serum endotoxin (0.252 EU/mL vs. 0.380 EU/mL at 21 days) and diamine oxidase (DAO; 0.819 U/mL vs. 3.952 U/mL at 21 days) levels following *E. coli* infection, indicating heightened intestinal permeability. Xu et al. (46) showed that necrotizing enteritis (NE) caused by *C. perfringens* infection led to reduced conductivity and short-circuit current values, along with increased fluorescein isothiocyanate–dextran flux, indicating heightened intestinal permeability in broilers. Furthermore, *C. perfringens* significantly downregulated the expression of Zonula Occludens-1 (*ZO-1*), *Claudin-1*, *Occluding*, and mucin 2 (*MUC-2*) genes in the small intestine of broilers, indicating that *C. perfringens* seriously damaged intestinal mucosal barrier function of broilers.

Numerous studies have shown that *C. butyricum* has a positive regulatory effect on the intestinal mucosal barrier of poultry (38, 46, 60, 61). *Clostridium butyricum* regulates the intestinal mucosal barrier of poultry mainly through the following ways:

Firstly, *C. butyricum* reduces intestinal permeability of poultry. Intestinal permeability, defined as the controlled passage of molecules across the intestinal epithelium, is a key determinant of barrier functions (17). An increased intestinal permeability can lead to increased translocation of pathogens and toxins, resulting in inflammation, impaired growth performance, and higher susceptibility to diseases (62). Therefore, understanding the factors that influence intestinal permeability and identifying effective strategies to maintain its integrity are of utmost importance in the poultry industry. *Clostridium butyricum* is a potential probiotic, and previous studies have provided convincing evidence that *C. butyricum* has a positive effect on intestinal permeability in poultry. For instance, Zhang et al. (38) demonstrated that the dietary inclusion of *C. butyricum* led to a notable reduction in endotoxin (0.380 EU/mL vs. 0.0.304 EU/mL at 21 days) and DAO levels (3.952 U/mL vs. 2.419 U/mL at 21 days), suggesting an amelioration of the intestinal mucosal damage caused by *E. coli*. Xu et al. (46) showed that dietary addition with *C. butyricum* led to increased conductivity and short-circuit current values, along with a decreased fluorescein isothiocyanate–dextran flux in intestinal of broilers infection with *C. perfringens*, which means that *C. butyricum* decreased intestinal permeability in broilers. A decrease in intestinal permeability means an enhanced intestinal barrier function, which can more effectively prevent harmful substances (such as bacteria, toxins, and incompletely digested proteins) from entering the bloodstream while promoting the absorption of nutrients, improving feed conversion rates and reducing mortality (63, 64).

Secondly, *C. butyricum* enhances the expression of intestinal tight junction proteins in poultry. Tight junctions, including ZO-1, Claudin-1, Occluding, are essential components of the intestinal mucosal barrier that protect against harmful pathogens (22). Previous studies have shown that *C. butyricum* can enhance the expression of intestinal tight junction protein in poultry, so as to maintain the integrity of the physical barrier and ensure its normal function (36, 60, 61, 65–67). For instance, Zhao et al. (60) showed that treatment with *C. butyricum* significantly upregulated the expression of intestinal *ZO-1* gene in *Salmonella enteritis*-infected broilers (6 days post-infection) and in Chicken intestinal epithelial cells (IECs) after 6 h post-infection with *Salmonella enteritis*. Li et al. (65) showed that dietary supplemented with *C. butyricum* (1 × 10^9^ cfu/kg) for 42 days significantly upregulated the expression of *claudin-1* and *ZO-1* mRNA in ileum of broilers.

Thirdly, *C. butyricum* stimulates the differentiation of intestinal goblet cells and promote the secretion of intestinal mucins (MUCs) (60, 68). For example, Liu et al. (36) showed that feeding 200 mg/kg, 400 mg/kg and 600 mg/kg *C. butyricum* for 42 days could significantly increase intestinal *MUC2* gene expression in Pekin ducks. Zhao et al. (60) showed that *C. butyricum* could alleviate intestinal damage of *Salmonella enteritis*-infected broilers by increasing the expression of *MUC-2* mRNA. Xu et al. (68) showed that broilers fed with *C. butyricum* (1 × 10^9^ cfu/kg) for 28 days significantly increased the number, length and density of goblet cells, as well as upregulated the expression of *MUC-2* mRNA in in ileum of broilers.

### *Clostridium butyricum* strengthens intestinal immune function and alleviates intestinal inflammation

The intestinal epithelium serves as not only a mechanical barrier in the intestine but also as one of the most crucial innate immune barriers. It plays a vital role in maintaining intestinal immune function by producing immune-related molecules and participating in the interaction of immune cells (22, 69). Within the intestinal epithelial mucosa, the lamina propria includes Peyer’s patches (PP), immune cells, antimicrobial peptides and secretory immunoglobulin A (sIgA), which are involved in protecting the host from invasion by foreign pathogens and maintaining the stability of the intestinal environment (22, 69–71). Many factors, such as heat stress (72) and pathogen infection (73), can cause intestinal inflammatory damage in poultry. Inflammatory response has a complex regulatory mechanism, and excessive inflammatory response will cause ir-reversible damage to the body. A large number of studies have confirmed that *C. butyricum* has an alleviating effect on intestinal inflammatory damage in poultry (38, 50, 65, 74).

When the intestinal mucosal barrier is damaged, Gram-negative bacteria may invade the intestinal mucosa, but the widespread presence of sIgA, an immunoglobulin secreted by the intestinal mucosal plasma cells, can effectively resist its invasion and destruction by interacting with these bacteria, thus playing a role in protecting the intestinal mucosal barrier (75, 76). Clear evidence supports the fact that *C. butyricum* can enhance the secretion of sIgA in the intestines of various animals, including rabbits (48, 77) and broiler chickens (74, 78). This suggests that one of the mechanisms by which *C. butyricum* regulates the intestinal immune barrier in poultry is by promoting the secretion of sIgA, which is particularly important in poultry, as intestinal health is directly linked to overall growth performance and disease resistance.

Anti-inflammatory cytokines such as interleukin (IL)-10 and transforming growth factor *β* (TGF-β) have been shown to downregulate pro-inflammatory cytokine production and promote tissue repair and homeostasis (79–81). By enhancing the production of anti-inflammatory cytokines, *C. butyricum* plays a crucial role in mitigating excessive inflammatory responses within the intestinal tract (46). This modulation helps maintain a balanced immune environment, which is essential for the overall health and productivity of poultry. For instance, Xu et al. (46) showed that infection with *C. perfringens* significantly induced intestinal inflammatory response in broilers, and dietary supplementation with *C. butyricum* alleviated intestinal inflammatory damage by promoting the secretion of anti-inflammatory factors IL-10 and TGF-*β*. Similarly, Huang et al. (61) showed that *C. butyricum* promoted intestinal IL-10 secretion in broilers with necrotizing enteritis caused by *C. perfringens*. At the same time, *C. butyricum* also had inhibitory effect on proinflammatory cytokines such as IL-1β, IL-6 and tumor necrosis factor *α* (TNF-α) in poultry intestines (60, 82). For instance, Zhao et al. (60) and Zhang et al. (83) showed that *C. butyricum* significantly inhibited the production of pro-inflammatory cytokines, such as IL-1β, IL-6, IL-8, interferon (IFN-*γ*) and TNF-*α*, thus alleviating the intestinal inflammatory damage caused by *Salmonella* in broilers. Similarly, Li et al. (65) showed that *C. butyricum* significantly decreased the expression levels of pro-inflammatory cytokines (IL-1β and TNF-α) to mitigate high stocking density-induced intestinal inflammatory damage in broilers. Although the immunomodulatory mechanisms of *C. butyricum* are general, their effects may be species-specific. That’s because the composition of the intestinal microbiota varies among different poultry breeds, due to the differences in genetic background, physiological characteristics and rearing methods (84), which can affect the colonization of exogenous microorganisms (such as *C. butyricum*) in the intestinal tract. For example, a certain type of *C. butyricum* may be more easily established in specific poultry breeds, producing more butyrate and thus exerting stronger immune-modulatory effects (85).

Studies have shown that *C. butyricum* can interact with related inflammatory signaling pathways to mitigate inflammatory pathological damage of poultry. For example, *C. butyricum* can inhibit toll-like receptor 4 (TLR4) signaling pathway, thus playing a protective role in Salmonella-induced intestinal inflammation of broilers (60, 86). Activation of nuclear factor-kappa B (NF-κB) signaling pathway induces the secretion of IL-1β, IL-8, TNF-*α* and IFN-*γ* and aggravates inflammation (87, 88). However, by inhibiting NF-κB signaling pathway, *C. butyricum* can reduce the levels of pro-inflammatory cytokines (IFN-γ, IL-1β, IL-6, IL-8, TNF-*α*), thereby reducing the inflammatory response of poultry (38, 60, 65, 86). For instance, Li et al. (50) showed that *C. butyricum* alleviated intestinal inflammatory response in broilers by inhibiting the activation of NF-κB, which was manifested as decreased expression of TNF-*α* and increased expression of anti-inflammatory cytokine IL-10. Zhao et al. (60, 86) showed that *C. butyricum* decreased the expression of pro-inflammatory factors (IFN-*γ*, IL-1β, IL-8, TNF-*α*) by inhibiting the TLR4-myeloid differentiation factor 88 (MyD88)-NF-κB pathway, thereby alleviating the intestinal inflammatory response of broilers caused by Salmonella infection. In summary, *C. butyricum* can reduce the expression of pro-inflammatory factors (TNF-α, IL-1β, IL-6 and IL-8), and promote the secretion of anti-inflammatory factors (IL-10 and TGF-β1) and immunoglobulin (sIgA) by inhibiting the TLR4-MyD88-NF-κb pathway, thereby reducing intestinal inflammation in poultry.

### *Clostridium butyricum* regulates intestinal microbial balance

The intestinal microbiota of poultry is a complex ecosystem that consists of a diverse array of microorganisms, along with their metabolites, which form the intestinal microbial barrier (17). The intestinal microbiota of poultry is predominantly composed of Firmicutes, Bacteroides and Proteobacteria, which primarily inhabit the intestinal mucosa surface and play a pivotal role in intestinal nutrient metabolism, permeability, and immunity (89, 90). Under normal physiological conditions, the probiotics and pathogenic bacteria in the intestinal environment of poultry are in a dynamic balance state, but this balance is easily affected by exogenous and endogenous factors (91, 92), which would lead to a variety of diseases and cause great economic damage to poultry production. Thus, the maintenance of intestinal microbial dynamic balance is of great significance for promoting healthy poultry breeding.

*Clostridium butyricum* have significant effect in regulating the intestinal flora of poultry. It can stimulate the growth of beneficial bacteria (such as *Lactobacillus* and *bifidobacterium*) inherent in the intestinal tract of poultry, and inhibit the reproduction of harmful bacteria (Such as *Salmonella*, *E. coli*, *Campylobacter*, *C. perfringens*) (37, 93–95), thereby reducing dysentery in poultry. The probiotic effect of *C. butyricum* on poultry is attributed to its strong ability to produce short-chain fatty acids (SCFAs), particularly butyric acid, which can reduce intestinal pH and inhibits the growth of harmful bacteria thereby improving the structure of intestinal flora (36, 47, 49, 96–98). Additionally, butyric acid can stimulate the expression of the *AVBD-9* gene in macrophages, bone marrow cells, and monocytes of poultry, and enhance the expression of host defense peptide genes such as *AVBD-9*, *AVBD-10*, *AVBD-14*, and *Cath B1* in poultry gut, effectively suppressing the invasion of foreign microorganisms (99, 100). Furthermore, *C. butyricum* itself, as a probiotic, can compete with pathogenic microorganisms for adhesion sites, thereby inhibiting their adhesion and colonization in the gut (17, 101). For instance, Yang et al. (37) showed that dietary supplementation with 2 × 10^7^ CFU/kg or 3 × 10^7^ CFU/kg of *C. butyricum* significantly reduced the number of harmful bacteria (*E. coli*, *Salmonella* and *C. perfringen*) and increased the number of beneficial bacteria (*C. butyricum*, *Lactobacillus* and *Bifidobacterium*) in ileum of broilers.

*Clostridium butyricum* can also improve intestinal microbial diversity, regulate intestinal microbial structure, and maintain intestinal microbial dynamic balance. For instance, previous studies have demonstrated that dietary supplementation of *C. butyricum* significantly influenced the intestinal microbial *α*-diversity, as evidenced by a significant increase in the ACE index, Chao 1 index and Shannon index (36, 45, 65). At the phylum level, *C. butyricum* supplementation could increase the proportion of Firmicutes and decrease the proportion of Proteobacteria in the intestinal tract of poultry (68, 82,). At the genus level, *C. butyricum* supplementation could increase the proportion of some beneficial bacteria, such as *Bacteroides* (65, 96), *Butyricicoccus* (68), *Lactobacillus* (45, 68) and *Barnesiella* (102), while reduce the proportion of some harmful bacteria, such as *Ralstonia* (45), *Escherichia-Shigella* (96) and *Klebsiella* (96, 103) in the intestinal tract of poultry. In conclusion, *C. butyricum* can enhance the diversity of intestinal microbiota in poultry, optimize the composition and structure of intestinal microbiota, thereby maintaining a dynamic balance and promoting a healthy intestinal environment in poultry.

## *Clostridium butyricum* promotes production performance of poultry

Intestinal health is very important for poultry growth and development. Optimal intestinal morphology and structure can facilitate the secretion of digestive enzymes, thereby enhancing the efficiency of nutrient digestion and absorption (38). A well-functioning intestinal barrier is essential for protecting poultry from various antigenic substances and ensuring their overall health and growth (104). Poultry is susceptible to heat stress (105), mycotoxin toxicity (106), and infections from pathogenic bacteria such as *Salmonella* (107), *E. coli* (108), and *C. perfringens* (109), which typically result in decreased feed intake and feed utilization efficiency, leading to a decline in overall production performance, thereby causing significant economic losses in the poultry industry. Many studies have shown that *C. butyricum* has a promoting effect on poultry production performance, including improving growth performance and product quality (Table 1).

**Table 1:** Production performance of poultry promoted by of *Clostridium butyricum*.

Poultry	Optimal added amount	Experimental period	Production performance	References
Broiler chickens	1.0 × 10^9^ CFU/kg	42 days	FBW↑, ADG↑, F/G↓	Cao et al. (35)
Broiler chickens	2.0 × 10^7^ or 3.0 × 10^7^ CFU/kg	42 days	ADG↑	Yang et al. (37)
Broiler chickens	2.0 × 10^7^ CFU/kg	28 days	FBW↑, ADG↑	Zhang et al. (38)
Broiler chickens	2.0 × 10^8^ CFU/kg	22 days	ADG↑, FCR↓	Xu et al. (46)
Broiler chickens	1.0 × 10^9^ CFU/kg	40 days	no effects	Zhang et al. (49)
Broiler chickens	1.5 × 10^9^ CFU/kg (1–21 d); 5 × 10^9^ CFU/kg (22–42 d)	42 days	FBW↑, ADG↑, a^*^2_4h_↑, pH_24h_↑, drip loss↓, shear force↓	Li et al. (50)
Broiler chickens	1.0 × 10^9^ CFU/kg	42 days	FBW↑, ADG↑, FCR↓	Li et al. (65)
Broiler chickens	1.0 × 10^9^ CFU/kg	28 days	FBW↑	Xu et al. (68)
Broiler chickens	2.0 × 10^7^ CFU/kg	28 days	FBW↑	Zhang et al. (74)
Broiler chickens	2.5 × 10^7^ or 5.0 × 10^7^ CFU/kg	42 days	FBW↑, ADG↑, FCR↓	Cao et al. (93)
Broiler chickens	1.0 × 10^6^ CFU/kg	42 days	no effects	Han et al. (97)
Broiler chickens	1.0 × 10^9^ CFU/kg	42 days	ADFI↑, ADG↑	Zhao et al. (110)
Broiler chickens	2.5 × 10^8^ CFU/kg, 5 × 10^8^ CFU/kg, 1 × 10^9^ CFU/kg	42 days	ADG↑	Liao et al. (111)
Broiler chickens	2.5 × 10^8^ CFU/kg, 5 × 10^8^ CFU/kg, 1 × 10^9^ CFU/kg	42 days	meat quality↑, breast muscle yield↑, fatty acid composition↑	Liao et al. (112)
Broiler chickens	100 g/t (1.0 × 10^8^ CFU/kg)	42 days	FI↓, F/G↓	Liu et al. (113)
Broiler chickens	4 × 10^8^ CFU/kg	39 days	FBW↑, ADG↑,	Yang et al. (114)
Broiler chickens	1.0 × 10^9^ CFU/kg	42 days	ADG↑	Zhao et al. (115)
Broiler chickens	2.5 × 10^8^ CFU/kg	42 days	ADG↑, F/G↓	Takahashi et al. (116)
Laying hens	1.0 × 10^8^ CFU/kg	28 days	average egg weight↑, eggshell weight↑, tibia index↑	Huang et al. (34)
Laying hens	0.02%	84 days	mortality rate↓, average egg weight↑, egg mass↑, egg production rate↑, feed efficiency↑, eggshell quality↑, Haugh unit↑, thick albumen content↑, albumen height↑	Obianwuna et al. (44)
Laying hens	0.5 g/kg	40 days	ADFI↓, FCR↓, eggshell strength↓, yolk color↓, albumen CP↑	Xiang et al. (58)
Laying hens	1.0 × 10^9^ CFU/kg	63 days	laying rate↑, egg mass↑, feed/egg↓, albumen height↑, eggshell thickness↑	Wang et al. (80)
Laying hens	5.0 × 10^7^ CFU/kg	70 days	egg production rate↑, egg mass↑, eggshell strength↑	Zhan et al. (91)
Laying hens	0.02%	84 days	egg production rate↑, average egg weight↑, egg mass↑, ADFI↑, damaged eggs↓, eggshell quality↑, Haugh unit↑, thick albumen con-tent↑, albumen height↑	Obianwuna et al. (118)
Laying hens	0.9 g/kg (8.37 × 10^8^ CFU/kg)	56 days	FCR↓, yolk color↑	Wang et al. (119)
Laying hens	1.0 × 10^9^ CFU/kg	56 days	egg mass↑, pre-grade yellow follicle number↑, yolk properties↑, albumen height↑, Haugh unit↑	Wang et al. (120)
Laying hens	2.5 × 10^9^ CFU/kg	56 days	ADFI↓, feed to egg ratio↓, eggshell ratio↑	Liu et al. (121)
Pekin ducks	400 mg/kg	42 days	ABW↑, ADG↑, F/G↓	Liu et al. (36)
Pekin ducks	200, 400, or 600 mg/kg	42 days	ADG↑, F/G↓, meat quality↑	Liu et al. (122)
Muscovy ducks	1 mL (2 × 10^9^ CFU/mL)	3 days	no effects	Xiao et al. (96)
Cherry Valley ducks	1.0 × 10^9^ CFU/kg	42 days	no effects	Zhang et al. (123)
Goose	250 mg/kg (3.0 × 10^6^ CFU/g)	70 days	ADG↑	Yu et al. (45)

ABW, average body weight; ADFI, average daily feed intake; ADG, average daily gain; FBW, final body weight; FCR, feed conversion ratio; F/G, feed intake to gain ratio; FI, feed intake. “↑” stands for increase, and “↓” stands for decrease.

From the summary table, it is evident that dietary addition of *C. butyricum* has a positive feeding effect on broiler chickens, laying hens, ducks and geese. In broilers, *C. butyricum* has been shown to increase average daily gain (ADG) and average daily feed intake (ADFI), improve feed conversion efficiency (decreased FCR or F/G), and improve meat quality and meat nutrient composition (35, 37, 38, 46, 49, 50, 65, 68, 74, 93, 97, 110–117). Similarly, for laying hens, *C. butyricum* has been found to improve laying rate, egg mass, average egg weight, and egg quality, while reducing the feed to egg ratio (34, 44, 58, 82, 95, 118–121). In the case of ducks, *C. butyricum* can increase ADG, decrease F/G, and improve meat quality (36, 122), but some studies believe that it has no effect on the production performance of ducks (96, 123). Although *C. butyricum* is less commonly used in goose production, limited studies suggest that it can increased the ADG of geese without affecting FCR (45). From this summary table, we can see that results for ducks and geese are less consistent than those for broilers or layers. This might be related to the fact that the digestive structure of ducks and geese is different from that of broilers and laying hens. Of course, the most important thing is that *C. butyricum* is used too little in ducks and geese. The limited data has a very significant impact on the accuracy of the conclusion, which requires further extensive research in the future with sufficient data support.

The promotion of poultry production performance by *C. butyricum* may be related to the following reasons: (i) it ferments and degrades undigested dietary fiber in the intestine to produce SCFAs, such as butyric acid (96–98), which directly supplies energy to intestinal epithelial cells, promotes intestinal villi growth, and enhances the digestion, absorption, and utilization of dietary nutrients in poultry, thus improving production performance. (ii) It stimulates the secretion of intestinal digestive enzymes (amylase, protease, lipase, and protease) (38, 49, 50) to break down macromolecular substances like carbohydrates, proteins, and lipids in the feed, thereby enhancing nutrient digestibility and improving feed conversion efficiency. (iii) It helps establish a healthy intestinal barrier for poultry (48, 60, 67, 100), which can effectively prevent the invasion of pathogenic substances and maintain the overall health of poultry, thereby improving the production performance of poultry.

Except for the addition of *C. butyricum* alone, the combined use of *C. butyricum* with other probiotics or functional substances has a synergistic promoting effect on the growth performance of poultry (47, 124). For instance, Zeng et al. (47) showed that dietary supplemented with a complex probiotic composed of *C. butyricum*, *B. subtilis*, and *Bacillus licheniformis* (*B. licheniformis*) can improve the overall health status of broilers, enhance their growth performance and immunity, and regulate the microbial community structure of the cecum. Zhang et al. (124) showed that dietary supplemented with a complex probiotic composed of *C. butyricum* and *B. subtilis* can effectively improve the growth performance and meat quality of broilers and regulate the microbial community structure in their excreta, which is of great significance to the poultry industry. However, it should be noted that the combined effect of probiotics may be influenced by multiple factors, including the type and dosage of probiotics, the breed of poultry, and the breeding environment. For instance, Yıldırım et al. (125) investigated the combined effects of tryptophan and probiotics on the growth and behavioral parameters of quail, it showed that supplementation with high-concentration tryptophan significantly reduced the weight gain of quails within 27 days, while probiotics had no obvious effect on this. Therefore, the combination of probiotics needs to be comprehensively considered in practical applications, and *C. butyricum* is no exception.

## Conclusion

In conclusion, *C. butyricum* serves as an effective regulator of intestinal health and exhibits a prebiotic effect on the growth of poultry. Dietary addition of *C. butyricum* promotes intestinal development, reduces oxidative damage, maintains intestinal barrier function, enhances intestinal nutrient digestion and absorption, improves feed efficiency, and ultimately enhances poultry production performance (e.g., increase weight gain and feed intake, increase egg production rate, and improve the quality of muscle and eggs). However, further research is still needed to fully elucidate the complex interactions and precise molecular mechanisms underlying its beneficial effects. Understanding these mechanisms in detail will not only provide valuable insights for optimizing poultry production but also contribute to the development of more effective and sustainable strategies in the poultry industry.
